# Visfatin aggravates transverse aortic constriction‐induced cardiac remodelling by enhancing macrophage‐mediated oxidative stress in mice

**DOI:** 10.1111/jcmm.17854

**Published:** 2023-08-16

**Authors:** Caijie Shen, Renyuan Fang, Jian Wang, Nan Wu, Shuangsuang Wang, Tian Shu, Jiating Dai, Mingjun Feng, Xiaomin Chen

**Affiliations:** ^1^ Department of Cardiovascular Medicine The First Affliated Hospital of Ningbo University, Key Laboratory of Precision Medicine for Atherosclerotic Diseases of Zhejiang Province Ningbo China; ^2^ Department of Cardiology Wenling First People's Hospital, The Affiliated Wenling Hospital of Wenzhou Medical University Wenzhou China; ^3^ Zhejiang University School of Medicine Hangzhou China; ^4^ Health Science Center, Ningbo University Ningbo China

**Keywords:** cardiac remodelling, macrophage polarisation, oxidative stress, transverse aortic constriction, visfatin

## Abstract

Previous studies have reported that visfatin can regulate macrophage polarisation, which has been demonstrated to participate in cardiac remodelling. The aims of this study were to investigate whether visfatin participates in transverse aortic constriction (TAC)‐induced cardiac remodelling by regulating macrophage polarisation. First, TAC surgery and angiotensin II (Ang II) infusion were used to establish a mouse cardiac remodelling model, visfatin expression was measured, and the results showed that TAC surgery or Ang II infusion increased visfatin expression in the serum and heart in mice, and phenylephrine or hydrogen peroxide promoted the release of visfatin from macrophages in vitro. All these effects were dose‐dependently reduced by superoxide dismutase. Second, visfatin was administered to TAC mice to observe the effects of visfatin on cardiac remodelling. We found that visfatin increased the cross‐sectional area of cardiomyocytes, aggravated cardiac fibrosis, exacerbated cardiac dysfunction, further regulated macrophage polarisation and aggravated oxidative stress in TAC mice. Finally, macrophages were depleted in TAC mice to investigate whether macrophages mediate the regulatory effect of visfatin on cardiac remodelling, and the results showed that the aggravating effects of visfatin on oxidative stress and cardiac remodelling were abrogated. Our study suggests that visfatin enhances cardiac remodelling by promoting macrophage polarisation and enhancing oxidative stress. Visfatin may be a potential target for the prevention and treatment of clinical cardiac remodelling.

## 1 INTRODUCTION

Cardiac remodelling is a complex clinical syndrome characterized by abnormal intrinsic changes in cardiomyocytes and the intercellular matrix. Cardiac remodelling is a key physiological process of chronic heart failure (CHF), which the World Health Organization predicts will cause more than 23 million deaths worldwide from 2002 to 2030.^1^ Although the survival rate of CHF has significantly improved, the overall prognosis is still poor, which places a great psychological burden on patients.^2^ Therefore, finding new interventions to delay the progression of cardiac remodelling is essential for the treatment of CHF.

Visfatin is a multifunctional adipokine that is widely expressed in a variety of tissues and organs.^3^ Visfatin was originally considered to be derived from adipocytes, was later discovered to be secreted by immune cells in adipose tissue, especially macrophages, and was recently found to be synthesized in macrophages in many other tissues and organs.^4^, ^5^, ^6^, ^7^ Inflammation has been reported to promote visfatin secretion, and recent studies have shown that oxidative stress promotes the synthesis and secretion of visfatin.^8^, ^9^, ^10^ Visfatin can regulate various biological effects, such as the inflammatory response, oxidative stress, cell differentiation, apoptosis and lipid metabolism.^10^, ^11^, ^12^, ^13^

Previous studies have shown that visfatin is involved in several cardiovascular diseases by modulating immune responses.^8^ A clinical study of acute myocardial infarction found that visfatin may contribute to plaque instability in coronary artery disease by promoting macrophage infiltration.^14^ Visfatin treatment increased aortic root macrophage infiltration and promoted MMP‐8 expression and plaque areas in high‐fat diet‐fed apolipoprotein E (ApoE)‐knockout mice.^15^ In doxorubicin‐treated mice, visfatin increased both CD3^+^ T lymphocyte and CD68^+^ macrophage infiltration and aggravated acute cardiac injury.^11^ Our recent study shown that visfatin amplified the cardiac inflammatory response in mice and exacerbated sepsis‐induced cardiac injury.^11^ However, whether visfatin is involved in cardiac remodelling has not been reported. In this study, we investigated whether visfatin plays a regulatory role in TAC‐induced cardiac remodelling and explored the possible mechanisms.

## 2 MATERIALS AND METHODS

### 2.1 Animal models and treatment

Male C57BL/6J mice aged 7–8 weeks were purchased from the Institute of Model Zoology of Nanjing University and fed in the animal house for 2 weeks. Then, mice weighing 25–27 g were selected and used in the experiments described below.

Part I: Mice underwent transverse aortic constriction (TAC) surgery or were infused with angiotensin II (Ang II, 1000 ng/kg/min) for 28 days to establish cardiac remodelling models. Some mice were intraperitoneally (i.p.) injected with different doses of the oxidative stress scavenger *N*‐acetyl‐cysteine (NAC, 125, 250 or 500 mg/kg) as described in a previous study.^16^ Mice underwent sham surgery or were infused with saline and were then treated with PBS as a control group (*n* = 5–10 in each group). Serum visfatin levels and cardiac visfatin mRNA expression in each mouse were detected.

Part II: WT mice underwent TAC surgery or sham surgery and were then treated daily with visfatin (100 μg/kg, Adipo Bioscience) or PBS (*n* = 10 in each group).^11^ Twenty‐eight days later, cardiac fibrosis, cardiac function, macrophage polarisation and oxidative stress in each group were measured.

Part III: TAC mice and sham mice were treated with clodronate liposomes (150 μL, FormuMax) every 3 days to deplete macrophages as described in a previous study,^17^ and some mice received the same amount of liposomes as the controls (*n* = 10 in each group). Then, all mice were analysed as described in part II.

This study was conducted according to the ‘Guide for the Care and Use of Laboratory Animals’ (NIH Publication No. 85–23, revised in 2010) and approved by the Laboratory Animal Welfare and Ethics Committee of Ningbo First Hospital.

### 2.2 Transverse aortic constriction and Ang II infusion

The mice were anesthetized by isoflurane inhalation at a concentration of 1.5%, ventilated by a ventilator, and placed flat on a heated operating table. Then, TAC surgery was performed as previously described.^18^ Briefly, the thoracotomy and intercostal space were dissected to reveal the aorta. A 7–0 nylon suture was passed through the aorta between the brachiocephalic trunk and the left common carotid artery and was tied around the aorta using a 27‐gauge needle. The nylon suture was constricted and tied, the needle was removed, and the thorax was closed. Sham‐operated mice underwent the same procedure, except that the sutures were not constricted and knotted. After returning to spontaneous breathing, all mice were weaned from the ventilator, given ketoprofen (5 mg/kg, Sigma) for analgesia and placed in an incubator at 28°C overnight. In addition, the hair between the shoulder blades on both sides was removed, and the skin was disinfected and cut open. A pouch approximately 1.5 cm × 4 cm in size was created after the needle holder was used to carefully separate the subcutaneous tissue. Then, a micro osmotic pump (Alzet, 2004 model) containing Ang II or saline was implanted into the pouch. After suturing the skin, the follow‐up procedure was the same as described above.

### 2.3 Cardiac ultrasound analysis

The preparation before cardiac ultrasound was performed as described previously. After shaving the hair around the left chest, the coupling agent was applied evenly to the area. A MyLab 30CV (Esaote) ultrasound system containing a 15 MHz probe was used to collect information on cardiac structure and function at different time points, including left ventricular end‐diastolic diameter (LVEDD), left ventricular end‐systolic diameter (LVESD), left ventricular posterior wall thickness (LVPWD), end‐diastolic ventricular septal thickness (IVSD), left ventricular ejection fraction (LVEF), and fractional shortening (FS). All data were collected from 15 cardiac cycles and averaged.

### 2.4 Cell studies and testing

Myocardial cells (MCs), cardiac fibroblasts (CFs) and macrophages (Møs) were used in this study, and all cells were cultured in RPMI‐1640 medium containing 10% foetal bovine serum (FBS) (both from Gibco). MCs and CFs were purchased from ATCC, while Møs were differentiated from monocytes induced by macrophage colony‐stimulating factor (M‐CSF, 50 ng/mL, PeproTech) for 8 days.^19^ After verification by flow cytometry, the purity of the Møs was approximately 92.9%. Monocytes were isolated as described in a previous study.^19^, ^20^ Briefly, male WT mice aged 6–8 weeks were euthanized by inhalation of 100% CO_2_. The tibia was separated, and both ends were cut open. All cells in the lumen were flushed out with 1640 medium. Erythrocyte lysis buffer was used to lyse erythrocytes, and monocytes with higher purity were obtained after being washed with PBS and centrifuged. The cells were treated as follows:

*Part I*: Møs were treated with phenylephrine (PE, 50 μM) or H_2_O_2_ (10 mM), and some of the cells were also treated with different doses of NAC (2.5 mM, 5 mM, 10 mM).^16^ Møs treated with saline and PBS were used as controls. Twenty‐four hours later, visfatin mRNA levels in Møs and visfatin levels in the medium were measured.
*Part II*: Møs (10^6^ cells) and MCs (10^6^ cells) were co‐cultured and divided into 8 groups as follows: 1: MCs + Møs; 2. MCs + Mø + VFT (100 ng/mL)^21^; 3. MCs + Mø + NAC; 4. MCs + Mø + VFT + NAC; 5: MCs + Møs + PE; 6. MCs + Mø + VFT + PE; 7. MCs + Mø + NAC + PE; and 8. MCs + Mø + VFT + NAC + PE. After 24 h, MCs were collected for ANP and BNP mRNA analysis.
*Part III*: Møs and CFs (10^6^ cells) were co‐cultured as described above, and the mRNA expression of α‐SMA, collagen I, and collagen III in CFs was measured.

### 2.5 Visfatin and oxidative stress detection

Blood samples were collected from the mice, and the cell culture supernatant was obtained. After centrifugation at 1000 × ***g*** for 20 min, the serum was collected. The expression levels of visfatin in the supernatant and serum were measured using mouse visfatin enzyme‐linked immunosorbent assay (ELISA) kits (Abcam) according to the manufacturer's instructions. In addition, serum superoxide dismutase (SOD) activity, glutathione (GSH) levels, NADPH oxidase activity, and malondialdehyde (MDA) levels were detected using appropriate kits (all purchased from Nanjing Jiancheng Bioengineering Institute) according to the manufacturer's protocols to assess oxidative stress levels.

### 2.6 Quantitative polymerase chain reaction (RT–qPCR)

Left ventricular tissue, Møs, MCs and CFs were extracted with TRIzol reagent, and total RNA was collected from each sample. Then, 2 μg of total RNA was used to synthesize cDNA using a reverse transcription kit (Roche). Forward primers, reverse primers, cDNA and LightCycler 480 SYBR Green Master Mix (Roche) were used to perform PCR amplification to detect target mRNA expression, including that of visfatin, atrial natriuretic peptide (ANP), B‐type natriuretic peptide (BNP), β‐myosin heavy chain (β‐MHC), transforming growth factor‐β (TGF‐β), connective tissue growth factor (CTGF), collagen Iα, collagen IIIα, inducible nitric oxide synthase (iNOS), CD38, CD80, CD86, arginase‐1 (Arg‐1), CD36, CD163, CD206 and α‐SMA. All mRNA levels were normalized to those of GAPDH, and the primer sequences used in this study are shown in Table S1.

### 2.7 Western blot analysis

Left ventricular tissue was lysed with RIPA lysis buffer and ultrasound, and total proteins were obtained from each sample and quantitated using a BCA protein kit (Thermo Fisher Scientific). Then, total proteins were separated by electrophoresis, transferred to Immobilon‐FL PVDF membranes (Millipore), and blocked with 5% nonfat milk. Then, the PVDF membranes were incubated with anti‐Nrf2, anti‐HO‐1, anti‐Nox2, anti‐Nox4 and anti‐GAPDH antibodies (all from Abcam or GeneTex) at 4°C overnight and incubated with secondary antibodies at room temperature for 1 h. Then, the target protein expression was scanned and analysed.

### 2.8 Histological analysis

The fixed and paraffin‐embedded hearts were cut to a thickness of approximately 5 μm and arranged on slides for subsequent analysis. Wheat germ agglutinin (WGA) staining was used to examine the morphology of the heart and analyse the cross‐sectional area (CSA) of cardiomyocytes. Masson staining was performed to measure collagen levels in left ventricular tissue. The heart tissue slides were incubated with anti‐F4/80, anti‐iNOS or anti‐Arg‐1 antibodies to measure the cardiac levels of total Møs, M1 macrophages (Mø1) and M2 macrophages (Mø2), respectively. A light microscope (Olympus) was used to acquire images from slides of Masson staining. The area of the blue signal was quantified, and the percentage of left ventricle fibrosis was calculated as the blue signal area divided by the total area. A fluorescence microscope system (TCS SP8, Germany) was used to obtain images from the slides that underwent WGA staining or immunofluorescence staining. The CSA information of more than 200 cardiomyocytes was collected from these slides and analysed in each group, and the average CSA represented the size of the cardiomyocytes in that group. For immunofluorescence staining, the intensity of the green fluorescence signal was quantified and analysed to represent the expression of cardiac iNOS or cardiac F4/80 in each group, while the red fluorescence signal represented the expression of cardiac Arg‐1.

### 2.9 Statistical analysis

The data in this study are presented as the mean ± SD and were analysed using GraphPad Prism 7. Differences in the means between two groups were compared using Student's *t*‐tests, and differences in the means among three or more groups were compared by anova, followed by Tukey's multiple comparisons test. A value of *p* < 0.05 was considered statistically significant.

## 3 RESULTS

### 3.1 Oxidative stress promotes visfatin expression during cardiac fibrosis

**FIGURE 1:**
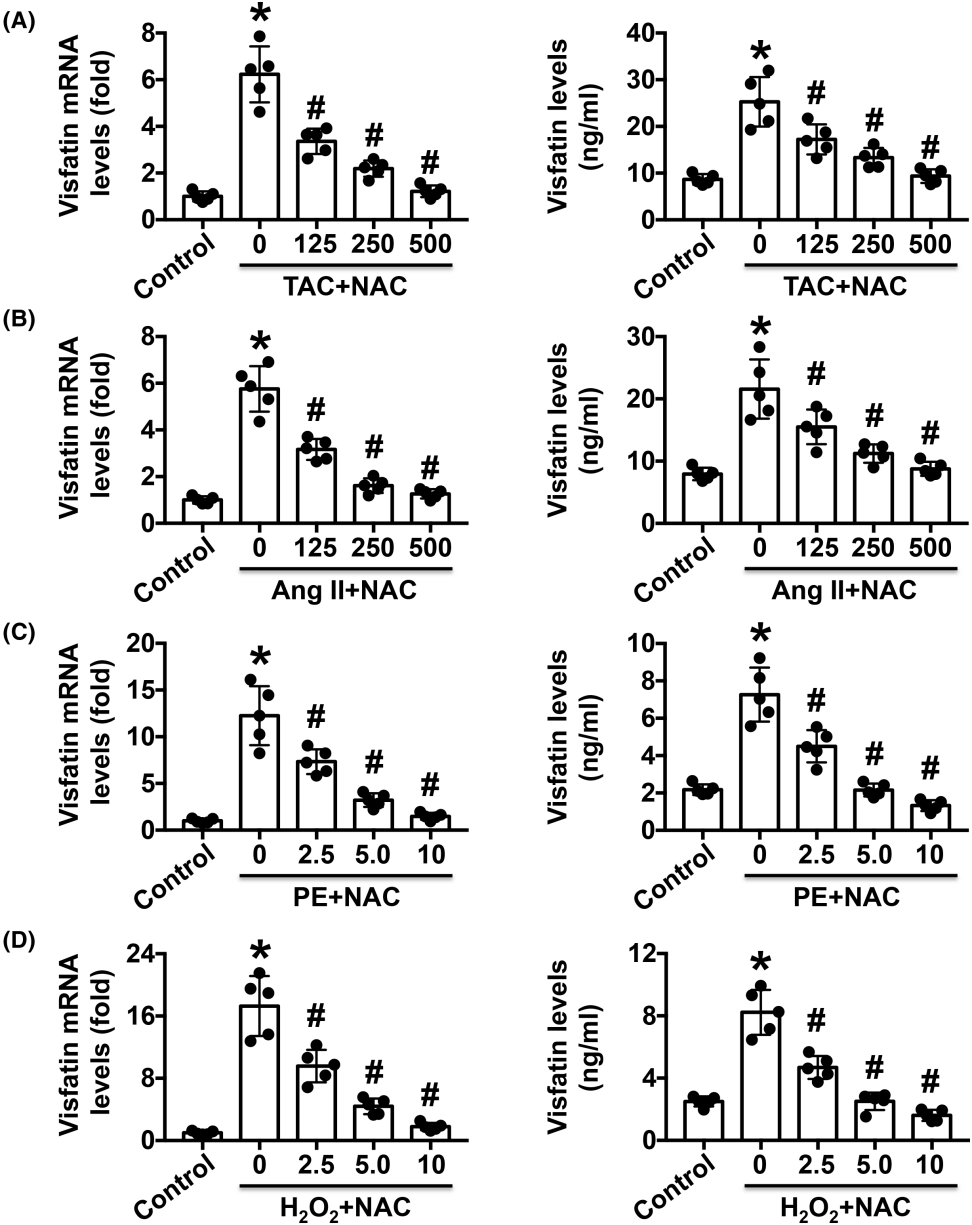
Effects of oxidative stress on visfatin expression during cardiac fibrosis. (A, B) Effects of NAC on visfatin expression in TAC‐ or Ang II‐induced mouse cardiac fibrosis models. (C, D) Effects of different doses of NAC on visfatin expression in PE‐ or H2O2‐treated Møs and the supernatant. *N* = 5 in each group. *p < 0.05 versus the control group. ^#^ *p* < 0.05 versus the previous group.

Visfatin expression was first measured in TAC‐induced and Ang II‐infused mice, and the results showed that cardiac visfatin expression and serum visfatin levels were significantly increased compared with those in control mice (Figure 1A,B). Dose‐dependent reductions in visfatin were observed after NAC treatment (Figure 1A,B). Similar changes in expression were found in PE‐ or H2O2‐treated Møs, and visfatin expression was also reduced by NAC (Figure 1C,D).

### 3.2 Visfatin aggravates TAC‐induced cardiac remodelling in mice

**FIGURE 2:**
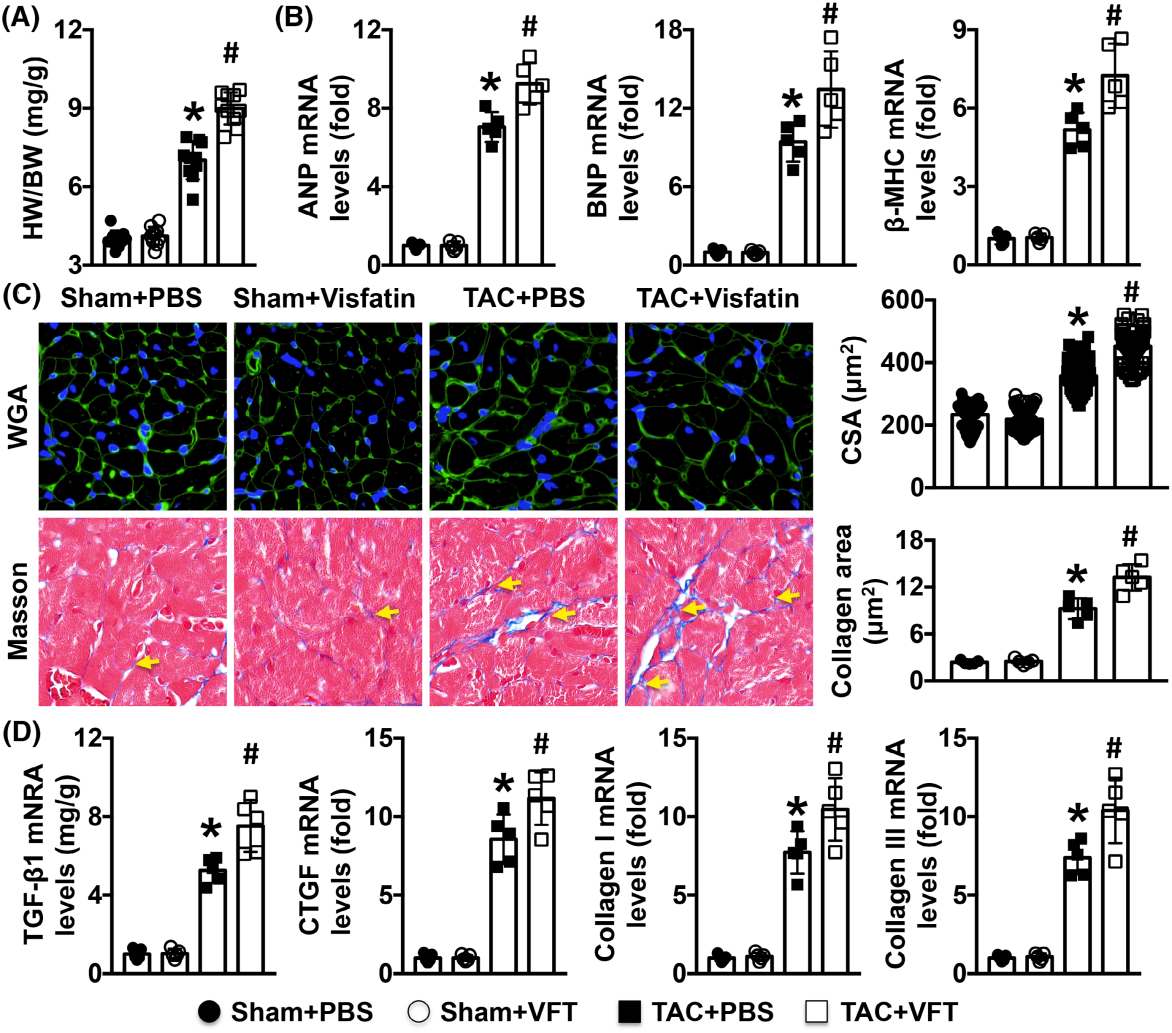
Effects of visfatin (VFT) on TAC‐induced cardiac remodelling in mice. (A, B) The heart weight/body weight (HW/BW) ratio and cardiac hypertrophy marker mRNA levels in the four groups were measured. (C) The CSAs of myocardial cells were measured by WGA staining (more than 200 cells were analysed), and the degree of cardiac fibrosis was determined by PSR staining. (D). The mRNA expression levels of TGF‐β1, CTGF, collagen I and collagen III were analysed. *N* = 5–10 in each group. *p < 0.05 versus the sham + PBS group. ^#^ *p* < 0.05 versus the TAC + PBS group.

**FIGURE 3:**
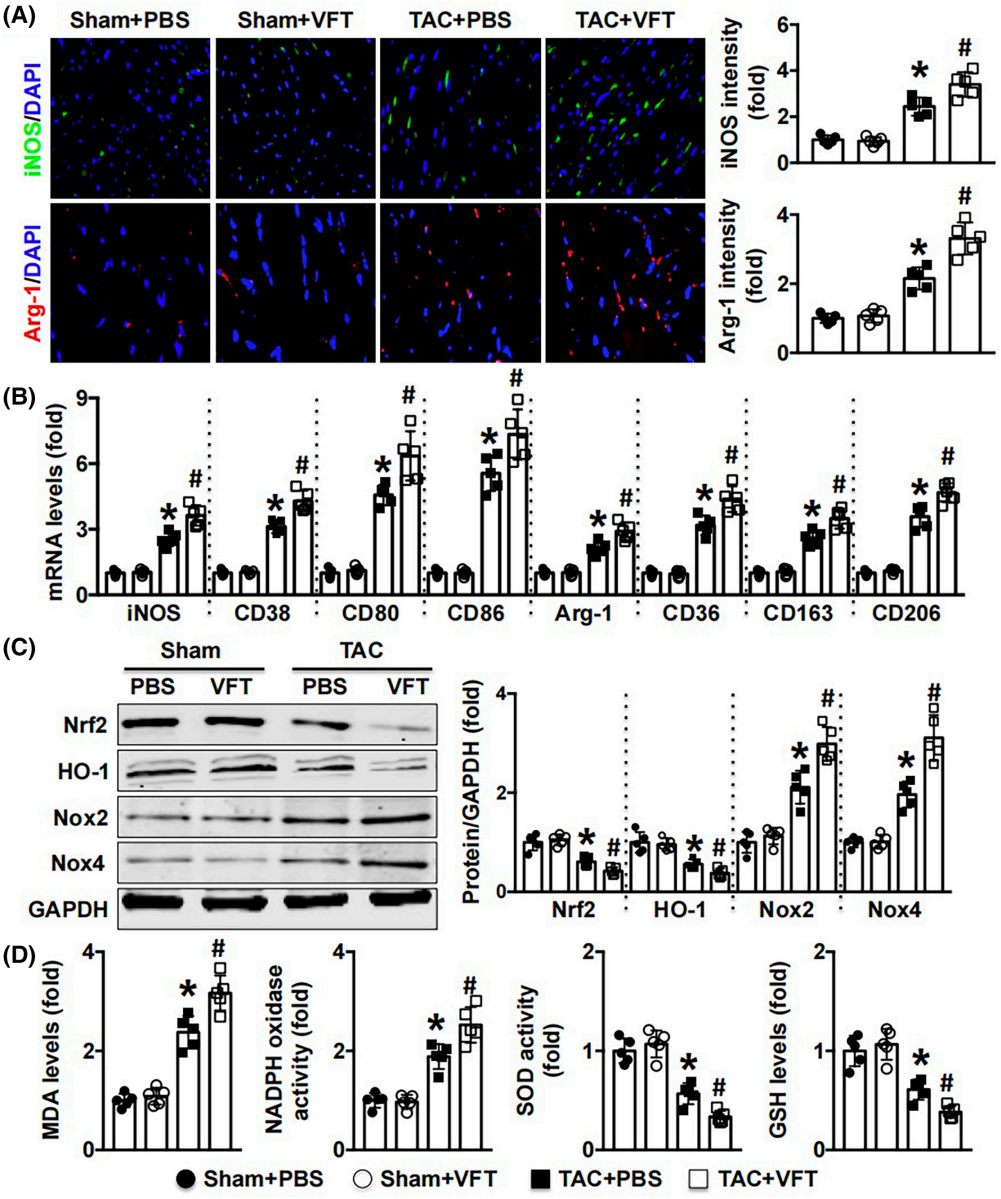
Effects of visfatin (VTF) on Mø‐related oxidative stress in TAC mice. (A). iNOS intensity and Arg‐1 intensity were measured by immunofluorescence staining. (B). Mø1 marker mRNA levels and Mø2 marker mRNA levels were detected. (C, D). Oxidative stress‐related pathways, MDA levels, NADPH oxidative activity, SOD activity, and GSH levels were detected. *N* = 5 in each group. *p < 0.05 versus the sham + PBS group. # *p* < 0.05 versus the TAC + PBS group.

Treatment with visfatin increased the ratio of heart weight (HW) to body weight (BW) in mice that underwent TAC surgery (Figure 2A). Higher mRNA expression levels of cardiac hypertrophy markers, including ANP, BNP and β‐MHC, were observed in cardiac remodelling mice (Figure 2B). Visfatin also further increased the CSA of MCs and collagen deposition in the left ventricle in mice that underwent TAC surgery (Figure 3C). Similar changes in TGF‐β1, CTGF, collagen Iα and collagen IIIα mRNA expression levels were observed (Figure 2D).

### 3.3 Treatment with visfatin worsens TAC‐induced cardiac dysfunction in mice

Treatment with visfatin further increased the LVEDD, LVESD, LVPWD and IVSD in mice that underwent TAC surgery (Figure S1 and Table S2). In addition, lower LVEF and FS levels were observed in the TAC + VFT group than in the TAC + PBS group (Figure S1 and Table S2).

### 3.4 Mø polarisation‐related oxidative stress is increased by visfatin in TAC‐subjected mice

Mø1‐ and Mø2‐related markers were first detected, and the results showed that treatment with visfatin enhanced iNOS intensity and increased the mRNA levels of iNOS, CD38, CD80 and CD86 in cardiac remodelling mice (Figure 3A,B). Visfatin also increased Arg‐1 intensity and elevated Arg‐1, CD36, CD163 and CD206 mRNA expression (Figure 3A,B). In addition, the antioxidative stress proteins Nrf2 and HO‐1 were reduced by visfatin, and oxidative stress proteins were increased (Figure 3C). Furthermore, treatment with visfatin increased MDA levels and NADPH oxidase activity in TAC mice but reduced SOD activity and GSH levels (Figure 3D).

### 3.5 Clodronate liposomes abolished the cardiac remodelling effect of visfatin on mice

**FIGURE 4:**
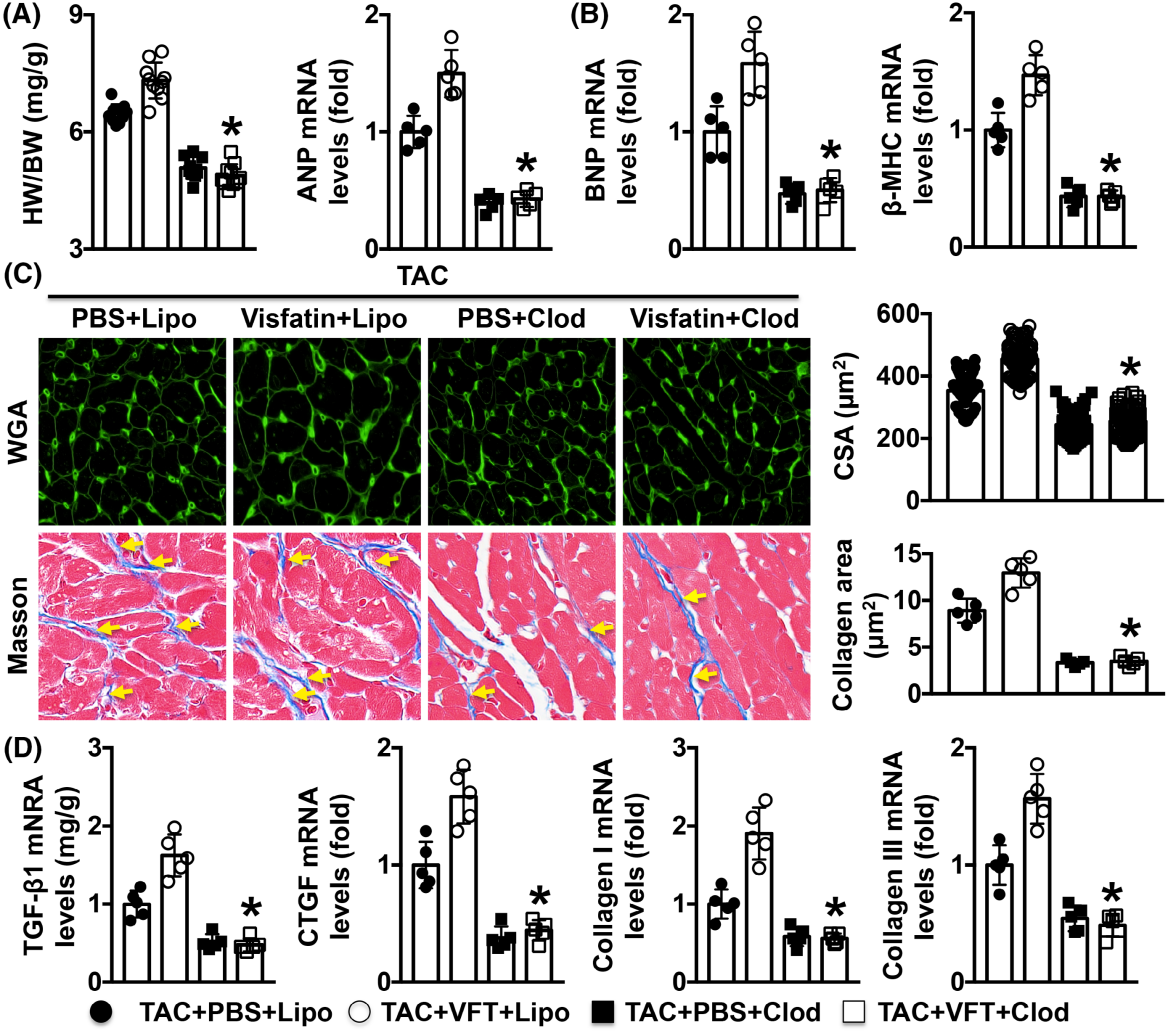
Effects of Mø depletion on TAC‐induced cardiac fibrosis in mice. (A, B). The HW/BW ratio and cardiac hypertrophy marker mRNA levels were measured. (C). The CSAs of myocardial cells and the degree of cardiac fibrosis were detected. (D). The mRNA expression levels of fibrosis markers were analysed. *N* = 5 in each group. *p < 0.05 versus the TAC + VFP + Lipo group. Clod, clodronate liposome; Lipo, liposome.

Treatment with clodronate liposomes or liposomes decreased the BWin TAC mice, but no differences in BW were found among the four groups. Treatment with clodronate liposomes decreased the HW/BW ratio and cardiac hypertrophy mRNA levels in TAC + VFT mice (Figure 4A). The HW and BW in each group are shown in Table S3. Treatment with clodronate liposomes decreased the HW/BW ratio and cardiac hypertrophy mRNA levels in TAC + VFT mice (Figure 4A,B). Clodronate liposomes also decreased the CSA of MCs and the deposition of collagen in visfatin‐treated TAC mice (Figure 4C). Decreased mRNA expression levels of TGF‐β1, CTGF, collagen Iα and collagen IIIα were observed in the TAC + VFT + Clod group compared with the TAC + VFT + Lipo group (Figure 4D). In addition, the LVEDD value was decreased and the LVEF was increased by clodronate liposomes in TAC + VFT mice (Figure S2 and Table S4).

### 3.6 Depletion of Møs alleviates oxidative stress in visfatin‐treated TAC‐subjected mice

**FIGURE 5:**
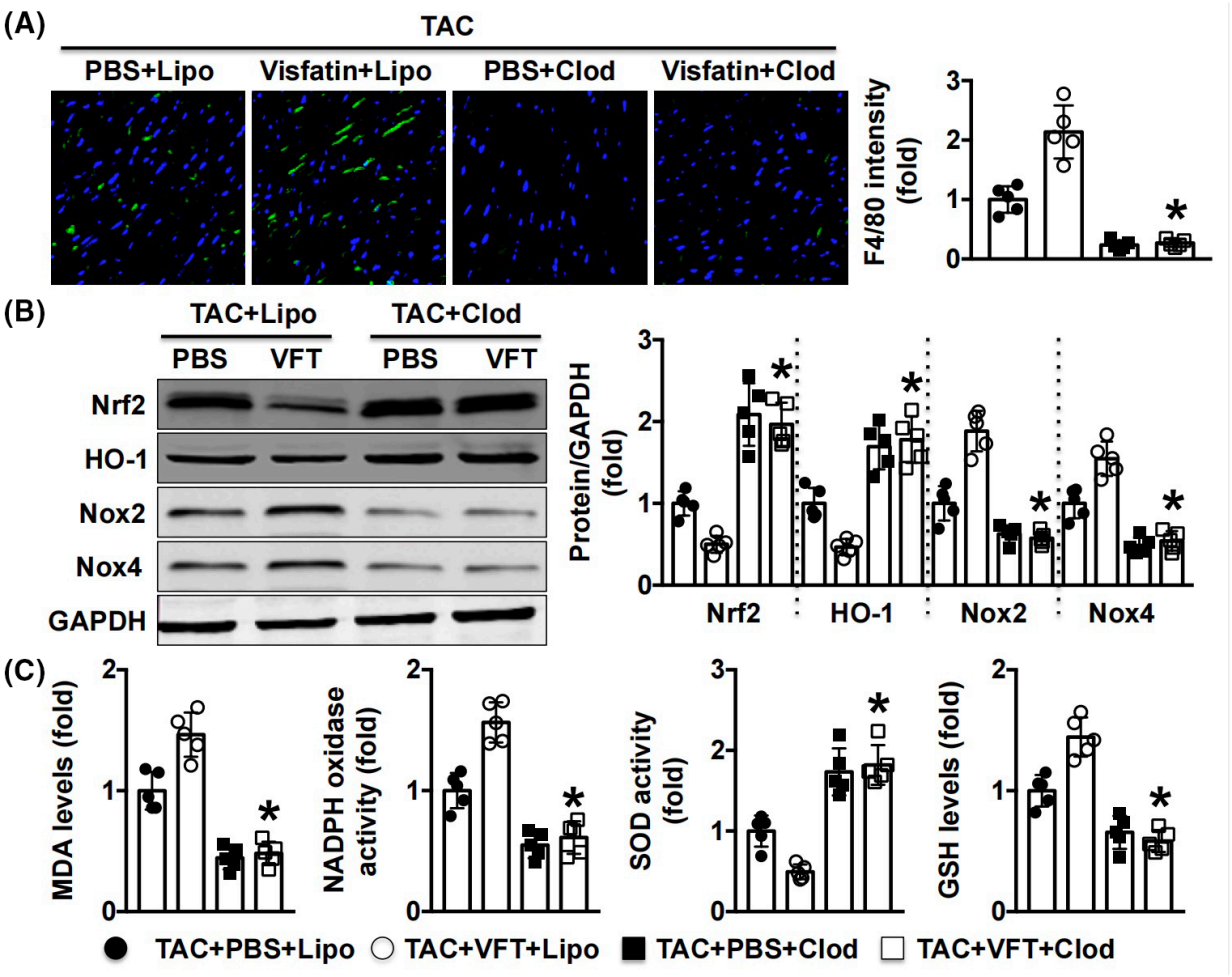
Effects of Mø depletion on Mø‐related oxidative stress in mice that underwent TAC surgery. (A). The F4/80 intensity was measured. (B, C). Oxidative stress‐related pathways and oxidative marker levels were detected. *N* = 5 in each group. *p < 0.05 versus the TAC + VFP + Lipo group. Clod, clodronate liposome; Lipo, liposome.

Immunofluorescence staining indicated that the depletion of Møs by clodronate liposomes significantly decreased F4/80+ cells compared with mice that received control liposomes (Figure 5A). Clodronate liposomes also increased Nrf2 and HO‐1 expression and decreased Nox2 and Nox4 levels in TAC mice (Figure 5B). Lower MDA levels and NADPH oxidase activity, as well as higher SOD activity and GSH levels, were found in TAC mice that received visfatin (Figure 5C).

### 3.7 Visfatin exacerbates PE‐induced MC hypertrophy and CF collagen deposition in vitro

**FIGURE 6:**
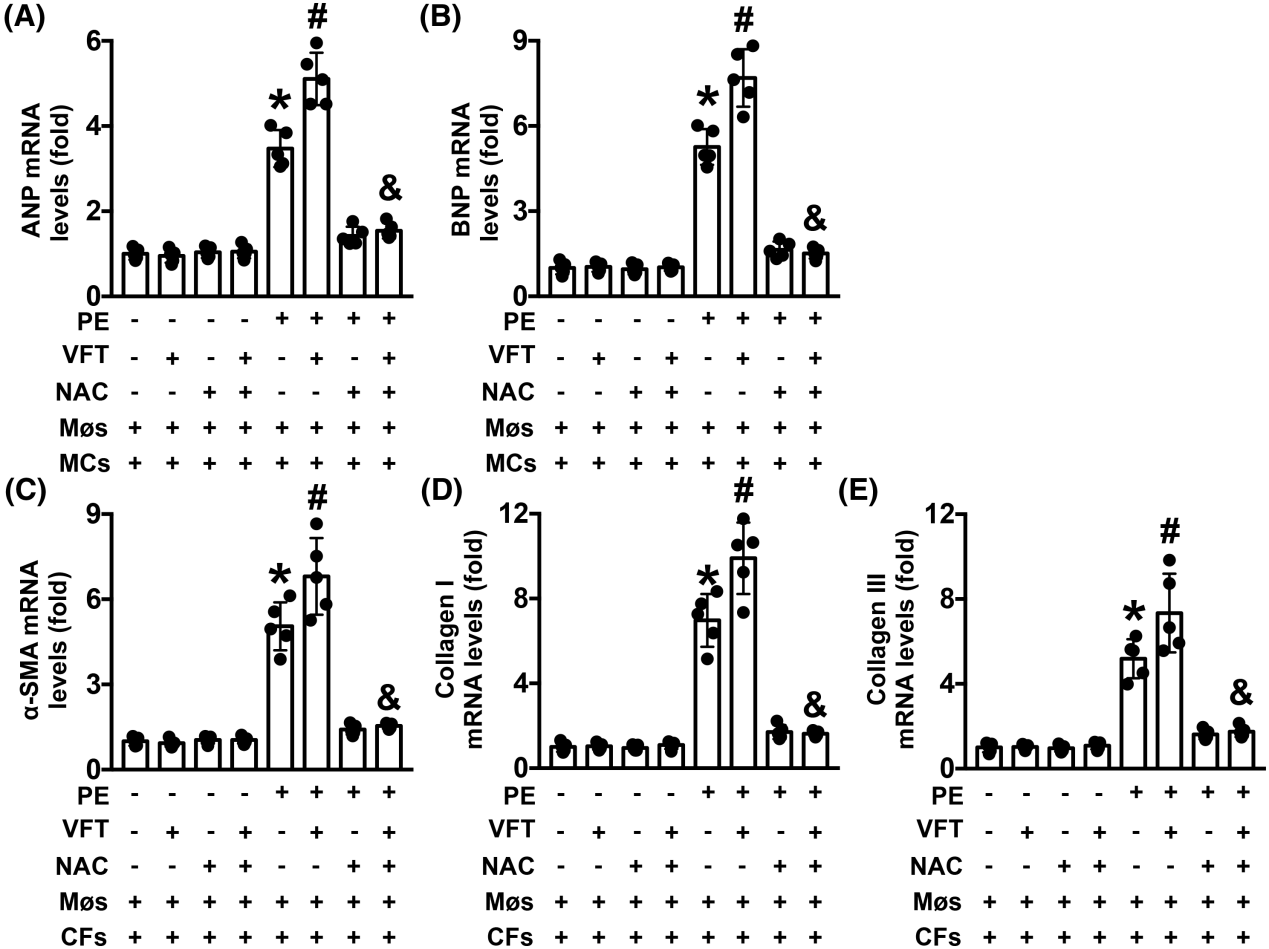
Effects of visfatin (VTF) and NAC on PE‐induced MC hypertrophy and CF collagen synthesis in vitro. (A, B). mRNA expression of ANP and BNP in MCs and (C–E). The mRNA levels of α‐SMA, collagen I and collagen III in CFs were measured. *N* = 5 in each group. *p < 0.05 versus the MCs/CFs + Mø group. ^#^ *p* < 0.05 versus the MCs/CFs + Mø + PE group. ^&^ *p* < 0.05 versus the MCs/CFs + Mø + PE + VFT group. VFT, visfatin.

When MCs were co‐cultured with Møs, ANP and BNP mRNA levels were increased by PE treatment and further elevated by visfatin (Figure 6A,B). These effects were reversed by NAC (Figure 6A,B). Similar changes in α‐SMA, collagen I and collagen III mRNA levels were found when CFs were cocultured with Møs (Figure 6C,E).

## 4 DISCUSSION

**FIGURE 7:**
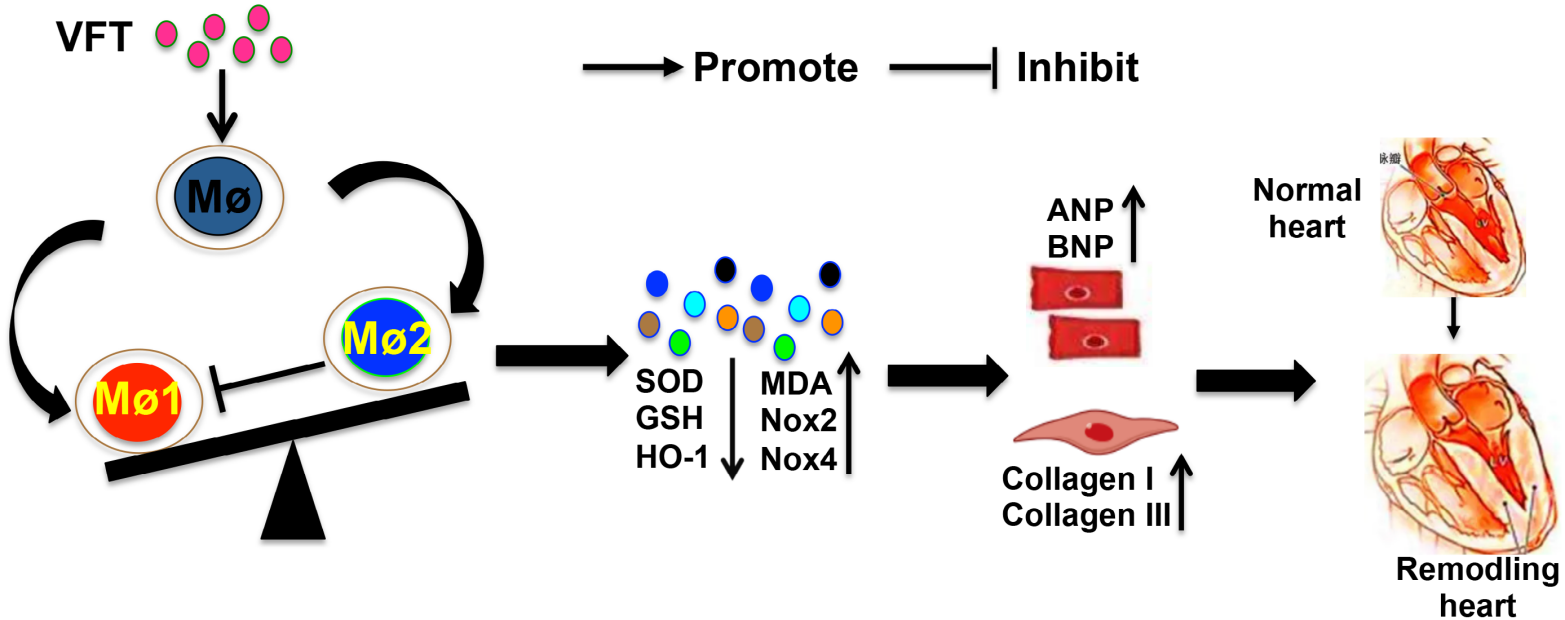
Mechanisms by which visfatin regulates cardiac remodelling.

In this study, we investigated the role of the adipokine visfatin in cardiac remodelling and explored the possible molecular mechanisms. In our study, we found that visfatin expression was increased in mice with cardiac remodelling, and PE and H_2_O_2_ promoted visfatin expression in vitro, which was reversed by the oxidative stress scavenger NAC. Exogenous visfatin treatment significantly promoted cardiac fibrosis, aggravated TAC‐mediated Mø polarisation and amplified oxidative stress. After the depletion of macrophages, the regulatory effects of visfatin on Mø‐related oxidative stress and cardiac fibrosis were abolished. These findings suggest that oxidative stress promotes visfatin release in pathological cardiac fibrosis and that visfatin participates in the progression of cardiac fibrosis by regulating Mø polarisation‐related oxidative stress (Figure 7).

In animal cardiac fibrosis models, increased infiltration and activation of a variety of immune cells can be observed, including monocytes/macrophages and lymphocytes.^22^, ^23^ After activation, these immune cells can release multiple cytokines or adipocytokines.^22^, ^23^ Immune cells, mainly Møs, have been shown to regulate the release of visfatin in previous studies.^11^ In this study, we examined the source and expression of visfatin in mouse models of cardiac remodelling. Our results showed that visfatin expression was significantly increased in TAC‐ or Ang II‐induced cardiac fibrosis models, and its expression was reversed by NAC. The expression of visfatin in Møs treated with PE and H_2_O_2_ was increased and reversed by NAC. These results indicate that visfatin is mainly produced by Møs and that its expression is regulated by oxidative stress. These findings also suggested that visfatin may participate in cardiac remodelling by regulating oxidative stress.

We next established a mouse cardiac remodelling model with TAC surgery and administered exogenous visfatin to determine whether visfatin regulates cardiac remodelling. The results showed that the CSAs of MCs, markers of cardiac hypertrophy and markers of cardiac fibrosis were significantly increased in TAC mice treated with visfatin. Echocardiography showed that cardiac hypertrophy and cardiac dysfunction were further worsened. These studies showed that visfatin aggravated cardiac fibrosis and cardiac dysfunction.

Although a variety of immune cells are involved in the process of cardiac remodelling, different types of immune cells have different regulatory effects on its progression. Among the many types of immune cells, Møs were found to be the most responsive to cardiac stress, followed by T and B lymphocytes.^24^ Furthermore, Møs also play the most important role in the process of cardiac adaptive remodelling to heart failure.^24^ A study confirmed that in addition to a few cardiac resident Møs, the vast majority of Møs in the reconstructed heart were infiltrated bone marrow‐derived Møs from the blood circulation.^24^ Although these are all Møs, the regulatory roles of cardiac resident Møs and bone marrow‐derived Møs in cardiac remodelling are not consistent.^24^, ^25^ A previous study showed that the process of cardiac remodelling was significantly accelerated after the depletion of cardiac resident Møs, suggesting that cardiac resident Møs inhibit the process of cardiac remodelling.^25^, ^26^, ^27^ Bone marrow‐derived macrophages can be involved in cardiac remodelling by differentiating into Mø1 and Mø2 cells.^28^ It was reported that the process of cardiac remodelling was significantly inhibited after all Møs were depleted by clodronate liposome treatment, suggesting that bone marrow‐derived Møs but not cardiac resident Møs play a leading role in the process of cardiac remodelling.^29^, ^30^

Mø1 and Mø2 cells play opposite roles, and Mø1 cells mediate tissue damage, while Mø2 cells mediate the repair of damaged tissue. Mø1 and Mø2 cells jointly mediate the occurrence and development of cardiac remodelling.^31^ Previous studies have confirmed that Mø1 polarisation is increased in the early stage of the pathological response in Ang II‐infused mice, while Mø2 polarisation is decreased. Then, Mø1 cells are maintained at a high level, while Mø2 cells are increased in response and are maintained at a parallel level with Mø1 cells, which suggests that Mø1 cells play a dominant role in pathological remodelling.^19^ To investigate the mechanism by which visfatin regulates cardiac remodelling, cardiac Mø polarisation was examined. The results showed that both Mø1 and Mø2 cells were significantly increased in mice subjected to TAC surgery for 28 days, which is consistent with previous results. However, after the depletion of macrophages, the regulatory effect of visfatin on cardiac remodelling was abolished, and cardiac dysfunction improved. These results suggest that visfatin aggravates cardiac remodelling and deteriorates cardiac function by regulating Mø polarisation.

Møs are some of the most important mediators of oxidative stress. Promoting Mø1 polarisation enhances cardiac oxidative stress and aggravates the process of cardiac remodelling, while inhibiting Mø1 polarisation alleviates cardiac oxidative stress and negatively regulates cardiac remodelling.^30^, ^31^ To determine whether visfatin participates in cardiac remodelling by regulating Mø polarisation‐related oxidative stress, cardiac Mø markers and cardiac oxidative stress levels were detected. The results showed that visfatin treatment significantly promoted Mø1 polarisation and aggravated cardiac remodelling in mice subjected to TAC surgery. In addition, the Nrf2 and HO‐1 antioxidant pathways were inhibited, and the expression of antioxidant proteins was reduced, while the Nox2 and Nox4 pro‐oxidant pathways were promoted, and the expression of pro‐oxidant proteins was increased. These findings indicate that visfatin amplifies cardiac oxidative stress. The effects of visfatin on cardiac oxidative stress and cardiac remodelling described above were reversed after Mø depletion. These results suggest that oxidative stress in cardiac remodelling is mainly mediated by Møs and that visfatin may promote Mø polarisation, further regulate oxidative stress‐related signalling pathways, promote the release of pro‐oxidants and inhibit the release of antioxidants in TAC‐subjected mice.

In conclusion, our study showed that oxidative stress encouraged Møs to release visfatin during cardiac remodelling. Visfatin promoted the polarisation of Møs induced by TAC surgery, amplified oxidative stress, and promoted the occurrence of cardiac remodelling and the deterioration of cardiac function. Visfatin may be a potential target for the clinical prevention of cardiac remodelling.

## AUTHOR CONTRIBUTIONS

**Caijie Shen:** Methodology (equal). **Renyuan Fang:** Methodology (equal). **Jian Wang:** Methodology (equal). **Nan Wu:** Writing – original draft (equal). **Shuangsuang Wang:** Writing – original draft (equal). **Tian Shu:** Methodology (equal). **Jiating Dai:** Methodology (supporting). **Mingjun Feng:** Project administration (equal). **Xiaomin Chen:** Project administration (equal).

## CONFLICT OF INTEREST STATEMENT

The authors declare no potential conflict of interest.

## Supporting information


Data S1.

## Data Availability

We confirm that all the data in our study could be freely available to scientists, except for commercial purposes.
