# Supplementary material for: Visfatin aggravates transverse aortic constriction‐induced cardiac remodelling by enhancing macrophage‐mediated oxidative stress in mice
Source: J Cell Mol Med. 2023 Aug 16;27(17):2562–71. doi: 10.1111/jcmm.17854 (PMC10468652; doi:10.1111/jcmm.17854)
Supplement: Supplementary file 1 — Data S1. [file JCMM-27-2562-s001.docx]

**Supplementary materials**

**Visfatin aggravates transverse aortic constriction-induced cardiac remodeling by enhancing macrophage-mediated oxidative stress in mice**

**
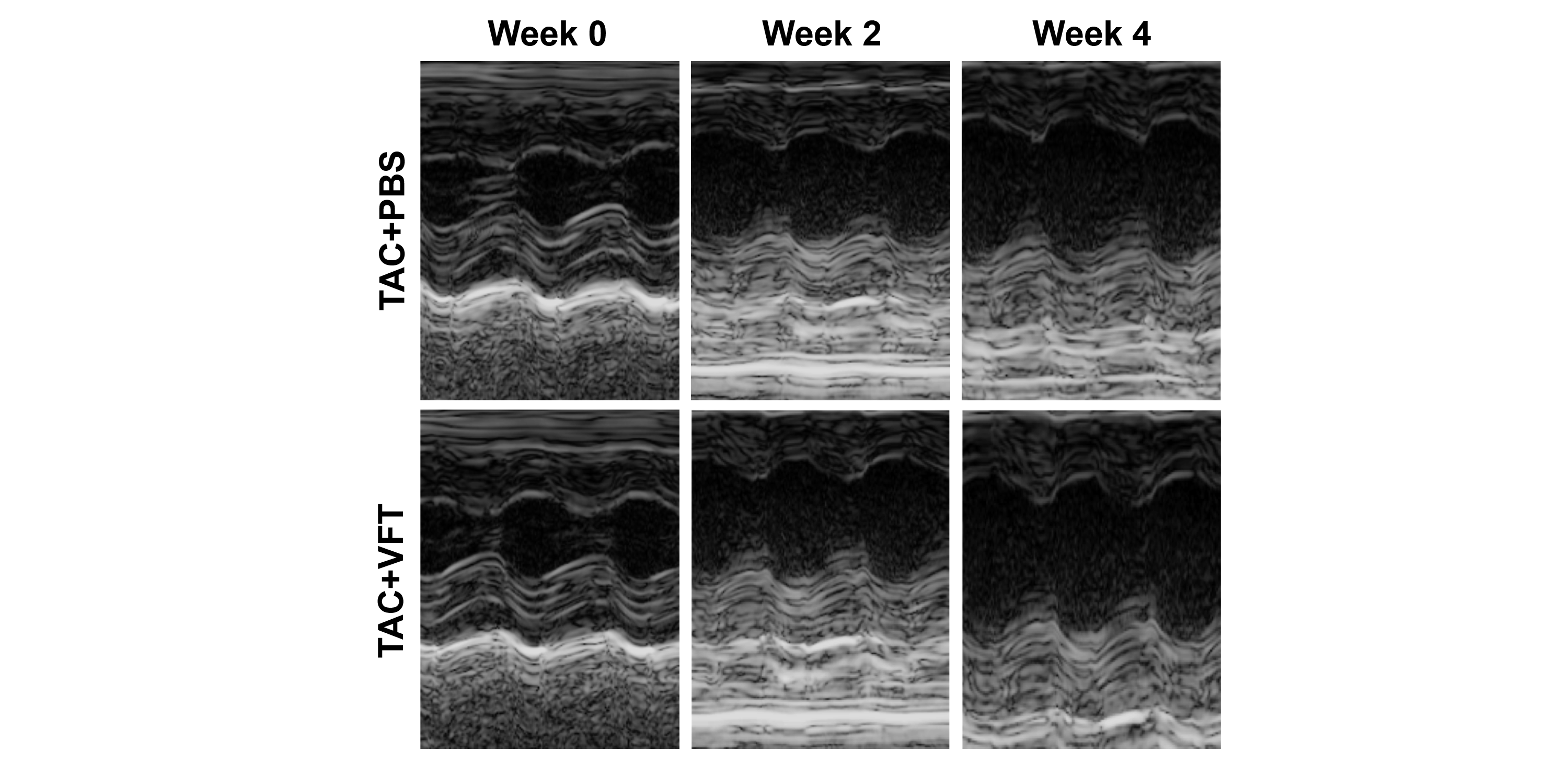
**

**Supplementary Figure 1:** Ultrasonographic images of mice underwent TAC surgery.


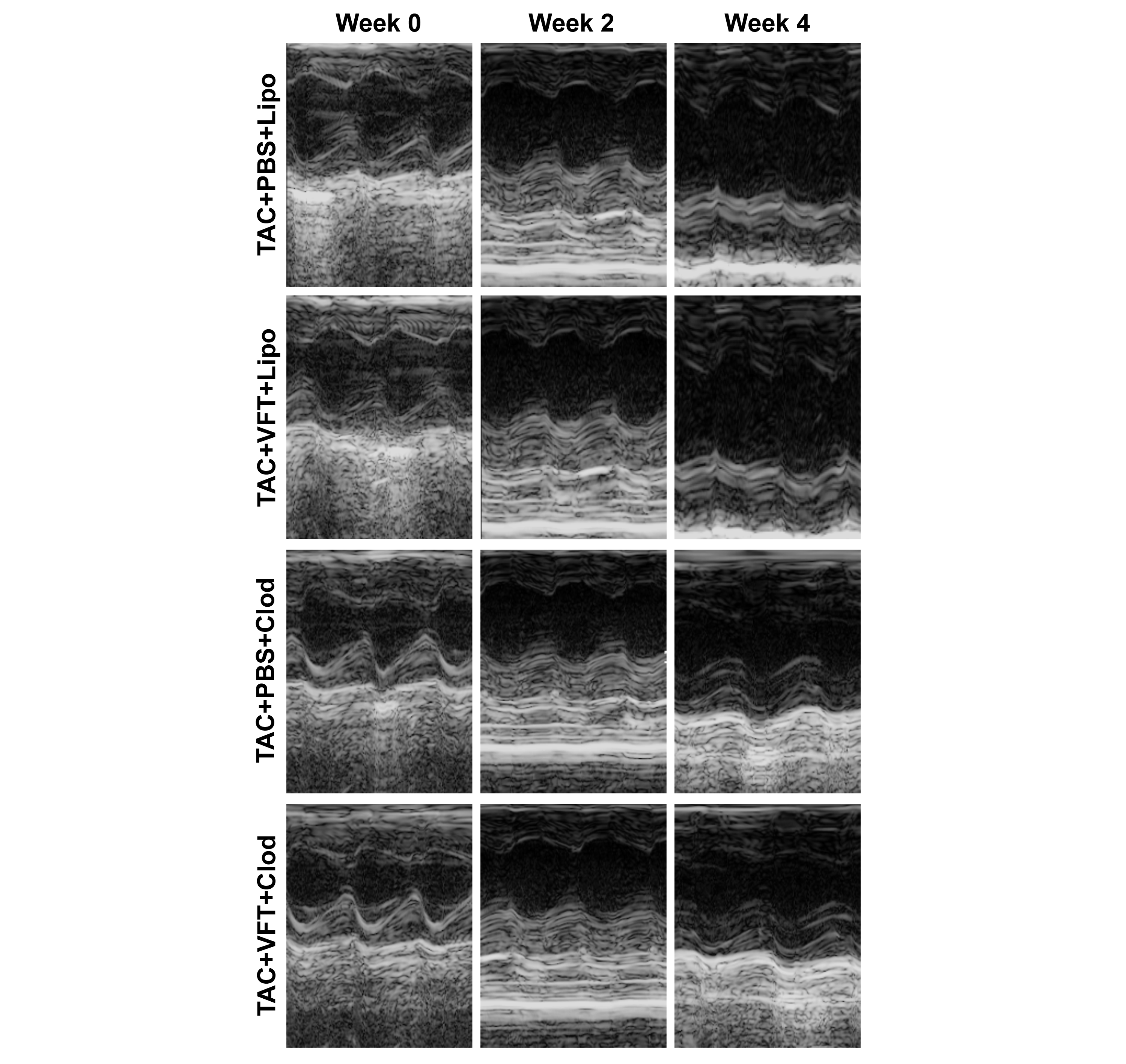


**Supplementary Figure 2:** Ultrasonographic images of mice treated with liposome or clodronate liposome.

.

**Supplementary Table 1: Primers used in this study**

| Genes | Forward primer (5′→ 3′) | Reverse primer (5′ → 3′) |
| --- | --- | --- |
| Visfatin | AATGTCTCCTTCGGTTCTGG | CCGCTGGTGTCCTATGTAAA |
| ANP | CCTGTGTACAGTGCGGTGTC | AAGCTGTTGCAGCCTAGTCC |
| BNP | CTCAAGCTGCTTTGGGCACAAGAT | AGCCAGGAGGTCTTCCTACAACAA |
| β-MHC | TCTACCCAGCCAAGATCAAAGT | CCCATTCCTAATAAGCTGTGTGG |
| TGF-β | TGTTAAAACTGGCATCTGA | GTCTCTTAGGAAGTAGGT |
| CTGF | TGACCCCTGCGACCCACA | TACACCGACCCACCGAAGACACAG |
| Collagen I | GCTCCTCTTAGGGGCCACT | CCACGTCTCACCATTGGGG |
| Collagen III | CTGTAACATGGAAACTGGGGAAA | CCATAGCTGAACTGAAAACCACC |
| iNOS | CGAAACGCTTCACTTCCAA | TGAGCCTATATTGCTGTGGCT |
| CD38 | TCTCTAGGAAAGCCCAGATCG | GTCCACACCAGGAGTGAGC |
| CD80 | GGCCTGAAGAAGCATTAGCTG | GAGGCTTCACCTAGAGAACCG |
| CD86 | GCTTCAGTTACTGTGGCCCT | TGTCAGCGTTACTATCCCGC |
| Arg-1 | AACACGGCAGTGGCTTTAACC | GGTTTTCATGTGGCGCATTC |
| CD36 | ATGGGCTGTGATCGGAACTG | TTTGCCACGTCATCTGGGTTT |
| CD163 | TCCACACGTCCAGAACAGTC | CCTTGGAAACAGAGACAGGC |
| CD206 | CAGGTGTGGGCTCAGGTAGT | TGTGGTGAGCTGAAAGGTGA |
| α-SMA | TCCTGACGCTGAAGTATCCGATA | GGCCACACGAAGCTCGTTAT |
| GAPDH | AACTTTGGCATTGTGGAAGG | CACATTGGGGGTAGGAACAC |

**Supplementary Table 2：Results of echocardiography in different time points.**

| Time | Group | LVEDD (mm) | LVESD (mm) | LVEF (%) | FS (%) |
| --- | --- | --- | --- | --- | --- |
| Week 0 | TAC+PBS | 3.44±0.15 | 2.59±0.19 | 76.6±1.9 | 43.1±2.1 |
|  | TAC+VFT | 3.48±0.16 | 2.61±0.21 | 76.4±2.2 | 43.1±2.5 |
| Week 2 | TAC+PBS | 4.74±0.23 | 3.28±0.27 | 62.4±2.8 | 35.2±1.7 |
|  | TAC+VFT | 5.07±0.25* | 3.68±0.27* | 59.1±2.3* | 32.4±2.5* |
| Week 4 | TAC+PBS | 5.11±0.25 | 3.62±0.28 | 54.1±3.1 | 29.3±2.4 |
|  | TAC+VFT | 5.47±0.21* | 4.08±0.31* | 48.9±2.5* | 26.4±1.8* |

**Supplementary Table 3：HW and BW in mice treated with Clodronate liposomes or liposomes.**

| TAC+PBS+Lipo | | TAC+VFT+Lipo | | TAC+PBS+Clod | | TAC+VFT+Clod | |
| --- | --- | --- | --- | --- | --- | --- | --- |
| HW (mg) | BW (g) | HW (mg) | BW (g) | HW (mg) | BW (g) | HW (mg) | BW (g) |
| 141 | 22.4 | 162 | 23.6 | 119 | 23.8 | 102 | 22.7 |
| 149 | 22.7 | 167 | 23.5 | 121 | 22.6 | 117 | 22.5 |
| 157 | 24.1 | 159 | 22.1 | 109 | 23.9 | 112 | 23.9 |
| 143 | 21.8 | 173 | 23.0 | 111 | 22.4 | 109 | 22.6 |
| 147 | 23.9 | 188 | 23.7 | 131 | 23.8 | 121 | 22.1 |
| 159 | 22.8 | 174 | 23.9 | 124 | 24.2 | 107 | 22.9 |
| 157 | 23.6 | 163 | 22.1 | 118 | 23.4 | 121 | 24.4 |
| 145 | 23.3 | 179 | 22.2 | 112 | 22.7 | 119 | 23.7 |
| 149 | 23.4 | 155 | 23.8 | 128 | 23.7 | 106 | 22.5 |
| 148 | 23.1 | 174 | 23.7 | 115 | 23.6 | 114 | 23.9 |

**Supplementary Table 4：Results of echocardiography in mice treated with Clodronate liposomes or liposomes.**

| Time | Group | LVEDD (mm) | LVESD (mm) | LVEF (%) | LVFS (%) |
| --- | --- | --- | --- | --- | --- |
| Week 0 | TAC+PBS+Lipo | 3.39±0.11 | 2.54±0.12 | 76.2±2.1 | 42.9±2.0 |
|  | TAC+VFT+Lipo | 3.34±0.10 | 2.58±0.17 | 77.1±2.4 | 43.3±2.4 |
|  | TAC+PBS+Clod | 3.36±0.15 | 2.61±0.21 | 76.9±1.8 | 43.1±2.2 |
|  | TAC+VFT+Clod | 3.48±0.16* | 2.57±0.19 | 77.0±2.1 | 42.8±2.3 |
| Week 2 | TAC+PBS+Lipo | 4.59±0.19 | 3.18±0.25 | 61.4±2.8 | 35.3±2.6 |
|  | TAC+VFT+Lipo | 5.03±0.22 | 3.56±0.22 | 58.2±2.6 | 32.9±2.0 |
|  | TAC+PBS+Clod | 3.78±0.20 | 2.74±0.22 | 72.3±3.1 | 40.8±2.4 |
|  | TAC+VFT+Clod | 3.65±0.16* | 2.84±0.23* | 70.2±2.3* | 39.5±1.9* |
| Week 4 | TAC+PBS+Lipo | 5.09±0.23 | 3.67±0.25 | 54.2±2.8 | 30.9±2.8 |
|  | TAC+VFT+Lipo | 5.42±0.26 | 4.12±0.31 | 48.1±2.6 | 27.4±2.8 |
|  | TAC+PBS+Clod | 3.90±0.22 | 3.02±0.21 | 70.0±3.5 | 39.8±2.6 |
|  | TAC+VFT+Clod | 3.82±0.24* | 3.08±0.24* | 69.8±2.7* | 39.2±1.9* |
